# Development of a microtiter plate-based assay for the detection of lipase-catalyzed transesterifications in organic solvents

**DOI:** 10.1007/s10529-014-1721-0

**Published:** 2014-11-12

**Authors:** Martin Pöhnlein, Tim Finkbeiner, Christoph Syldatk, Rudolf Hausmann

**Affiliations:** 1 Institute of Process Engineering in Life Sciences, Section II: Technical Biology, Karlsruhe Institute of Technology (KIT), Engler-Bunte-Ring 1, 76131 Karlsruhe, Germany; 2 Institute of Food Science and Biotechnology, Bioprocess Engineering, University Hohenheim, Stuttgart, Germany

**Keywords:** Assay, Glycolipid synthesis, Lipase, Organic solvents, Transesterification

## Abstract

A microtiter plate-based assay was developed to evaluate the ability of lipases to perform transesterifications when employed in different organic solvents. A 4-nitrophenol assay was carried out employing seven different lipase formulations and two fatty acid methyl esters with different chain lengths in a total of six organic solvents with logP values approximately between 1 and −1. This assay delivered results within comparatively short times measured by a color reaction and thus facilitates the choice of an enzyme-solvent combination for the synthesis of glycolipids. To validate the findings, glycolipid syntheses were performed using the same lipase formulation in the same solvents. When comparing the results obtained using the microtiter plate-based assay to the results of the glycolipid syntheses using the same lipases and solvents, matching results were obtained.

## Introduction

Lipases (E.C. 3.1.1.3, triacylglycerol acyl hydrolases) are hydrolases that act on ester bonds. They are widely used in many processes such as the synthesis of biodiesel (Korman et al. 2013) and monoacylglycerols (Bornscheuer 1995) or for the racemic resolution of different molecules (Öhrner et al. 1996). Since the late 1980s lipases have gained special interest due to their reverse hydrolytic and transesterification activity exhibited when employed in anhydrous reaction media (Klibanov 1989). Thus, lipases have been widely used to synthesize ester bonds; for example for the enzymatic synthesis of glycolipids, where different lipases in varying formulations have been used in several organic solvents (Coulon et al. 1996; Degn et al. 1999). When performing enzymatic glycolipid syntheses, the choice of solvent and catalyst are crucial parameters.

To evaluate the activity of lipases, several screening assays have been proposed (Amaya et al. 1995; Hasan et al. 2009) and have been carried out in aqueous solutions mainly focusing on the enzymes hydrolytic activity, which however is not always correlated to its synthetic activity (Sandoval and Marty 2007). Thus, in order to examine lipase activity in organic solvents, several assays have been developed or existing assays have been refined and modified to determine the lipases synthetic activities as well (Furutani et al. 1995; Pencreac’h and Baratti 1996). Usually these assays however are carried out in unpolar solvents like hexane or heptane (Goujard et al. 2009; Teng and Xu 2007). Although providing enzyme stability, these solvents are not suitable to monitor glycolipid syntheses, since sugars do not dissolve in them. To circumvent this problem a 4-nitrophenol (para-nitrophenol, pNP) assay has been developed to determine the synthetic activity of different lipases in organic solvents with a lower logP value ranging from approximately 1 to −1, which is described within this work.

## Materials and methods

### Materials

4-Nitrophenol, lipase B from *Candida antarctica*, immobilized on acrylic resin (Novozyme 435), lipase from *Rhizomucor miehei* immobilized on macroporous ion-exchange resin (Lipozyme), lipase from *Candida rugosa*, lipase from *R. miehei* and lipase from *Thermomyces lanuginosus* were purchased from Sigma-Aldrich. Lipase A (CalA) and lipase B (CalB) from *C. antarctica* (both lyophilized) were gifts from cLEcta (cLEcta GmbH, Leipzig, Germany). Methyl *n*-octanoate and methyl *n*-palmitate were purchased from Tokyo Chemical Industry Co. Ltd (TCI-Europe, Zwijndrecht, Belgium). The various solvents were the highest purity available and used without further treatment.

### Methods

#### pNP ester synthesis

Ten mM pNP solutions were prepared in a total of six solvents, which, listed with increasing polarity, were methyl *tert*-butyl ether (MTBE, logP value 1.18), 2-methyl-2-butanol (2M2B, logP value 1.06), tert-butanol (logP value 0.54), 2-propanol (logP value 0.25), acetone (logP value 0.11) and acetonitrile (ACN, logP value −0.17). Since *tert*-butanol tends to appear in solid state at room temperature all solutions were preheated to 40 °C. After addition of 10 mg or 10 µl of the enzyme formulation to be tested, the reaction was started by adding 60 mM methyl octanoate or methyl palmitate. The reaction took place in 2 ml vessels under constant shaking at 1,200 rpm and 40 °C with a total reaction volume of 1 ml. Samples were withdrawn after 0, 10, 20 and 30 min in order to determine the initial transesterification activity by following the depletion of pNP. Experiments were carried out with a total of seven different lipases, which were Novozyme 435 (Lipase B from *C. antarctica,* immobilized on acrylic resin), Lipozyme (Lipase from *R. miehei*, immobilized on macroporous ion-exchange resin), CalA (Lipase A from *C. antarctica,* lyophilized*),* CalB (Lipase B from *C. antarctica,* lyophilized*),* lipase from *T. lanuginosus* (liquid formulation), lipase from *R. miehei* (liquid formulation) and lipase from *C. rugosa* (lyophilized). When using the immobilized lipases, Novozyme 435 and Lipozyme, the enzyme was allowed to settle before taking a 5 µl sample from the reaction mixture, which was directly mixed with 250 µl Tris buffer (50 mM Tris/HCl, pH 7.0 + 0.1 % Triton X) in a microtiter plate. To extract the pNP, the microtiter plate was shaken for 10 s at 1,200 rpm, followed by the measurement of absorption at 412 nm in a microtiter plate reader. At 412 nm only the substrate pNP exhibits an absorption maximum while the resulting pNP esters show no absorption. Thus it is possible to monitor this reaction by following the decrease of absorption and subsequently calculating the pNP concentration and the resulting enzyme activity. Experiments using lipases in liquid or powdered formulations were treated as follows to avoid the transfer of enzymes to the aqueous phase. After 0, 10, 20 and 30 min, 100 µl samples were withdrawn into a 1.5 ml reaction vessel which was then centrifuged at 13,000 rpm for 1 min to precipitate free enzymes. 5 µl of the clear supernatant was then mixed with the Tris/HCl buffer and treated as described before. All experiments, including blanks without fatty acid esters or without enzymes, were carried out as triplets.

#### Glycolipid synthesis

18 mg (10 mM) of glucose were mixed with the corresponding solvent together with 100 mg or 100 µl of the lipase formulation to be tested. To start the reaction 60 mM methyl octanoate (94 µl) or methyl palmitate (162 µl) were added to give a total volume of 10 ml. The reaction was incubated for 48 h at 40 °C and 300 rpm in a shaking-water bath. All experiments were carried out as triplicates. Samples from each reaction were drawn at timed intervals to follow the proposed transesterification between glucose and the used fatty acid methyl ester.

#### Thin layer chromatography (TLC)

TLC was performed for qualitative analysis of the performed glycolipid synthesis. 10 µl samples were applied onto an analytical silica gel 60 TLC plate (10 × 20 cm, film thickness 0.25 mm). The plates were developed in chloroform/methanol/acetic acid (65: 15: 2 by vol.). Visualization was accomplished by dipping the plate in an anisaldehyde solution (anisaldehyde/sulfuric acid/acetic acid, 0,5: 1: 100, by vol.) followed by heating at 200 °C under constant air flow for about 5 min.

## Results and discussion

### Synthesis of pNP esters in organic solvents

If the tested lipase exhibited transesterification activity, it could be determined from the colorimetric reaction. Subsequent calculation of the initial transesterification activity then allowed the comparison between the different lipase—solvent combinations. All experiments were carried out with methyl octanoate and methyl palmitate, which results will be discussed separately.

#### pNP ester synthesis using methyl octanoate

To compare the initial transesterification activities of the different lipases employed in different solvents, the results obtained using pNP and methyl octanoate are shown in Table 1.

**Table 1:** Detected initial transesterification activities during the lipase-catalyzed synthesis of 4-nitrophenol esters using methyl octanoate

Lipase activity [nmol pNP/min]	MTBE	2M2B	t-Butanol	2-Propanol	Acetone	ACN
Novozyme 435	–	51.2 ± 33.3	93.6 ± 8.1	9.9 ± 5.3	27.6 ± 12.1	112.5 ± 21.2
Lipozyme	–	141.4 ± 31.6	51.1 ± 23.3	–	52.9 ± 22.1	31.8 ± 31.1
Cal A	–	64.2 ± 10.3	–	11.5 ± 0	–	–
Cal B	–	27 ± 8.4	–	–	–	–
Lipase *T. lanuginosus*	–	58.4 ± 18.1	5.8 ± 0.4	–	–	–
Lipase *R. miehei*	–	–	54.2 ± 46.5	–	–	16.7 ± 6.2
Lipase *C. rugosa*	–	25.9 ± 22.7	–	–	–	–

In approximately 40 % (17 out of 42) of all possible combinations of lipase and solvent a transesterification activity of the lipase could be detected. The use of Novozyme 435, for example, resulted in a transesterification in all tested solvents except for MTB, the use of Lipozyme was successful in all solvents, except MTBE and 2-propanol. For all other lipases, transesterification activities could only be measured in a maximum of two solvents. Possible explanations for the lack of activity of the lipases in certain solvents might be the polarity of the given solvent, and what might lead to the disruption of the water layer around the enzyme which is essential for its flexibility and activity (Gorman and Dordick 1992). Thus, lipase formulations, like Novozyme and Lipozyme, might exhibit higher transesterification activities since they are more stable in polar solvent due to their immobilization. Powdered or liquid lipase formulations oftentimes exhibited aggregation, especially when employed in more unpolar solvents, which might indicate enzyme denaturation, thus possibly explaining their lack of transesterification activity. Additionally, other factors such as the initial water content of the solvent might influence the enzymes activity as well.

The most non-polar solvent tested was MTBE, in which no activity could be observed for any lipases 2M2B and *tert*-butanol are less unpolar and seem to provide a good combination of substrate solubility and enzyme activity, thus showing transesterification activities for most of the lipases employed. In the more polar solvents 2-propanol, acetone and acetonitrile the immobilized lipases Novozyme 435 and Lipozyme could synthesize ester bonds between pNP and the fatty acid, but mostly with moderate activity. Only the combination of Novozyme 435 in acetonitrile presented a distinct exception, showing a very high transesterification activity. To sum up: the highest activities were measured when using Novozyme 435 in acetonitrile or *tert*-butanol and with Lipozyme in 2M2B, making these combinations interesting for an enzymatic glycolipid synthesis using methyl octanoate.

#### pNP ester synthesis using methyl palmitate

To investigate the influence of the fatty acid chain length and to find suitable enzyme-solvent combinations for transesterification reactions using long chain fatty acid esters, experiments, analogous to the ones with methyl octanoate, were carried out employing methyl palmitate. The results of these experiments are shown in Table 2.

**Table 2:** Detected initial transesterification activities during the lipase-catalyzed synthesis of 4-nitrophenol esters using methyl palmitate

Lipase activity [nmol pNP/min]	MTBE	2M2B	t-Butanol	2-Propanol	Acetone	ACN
Novozyme 435	84.9 ± 24.4	76.2 ± 28.5	48.5 ± 6.5	–	–	–
Lipozyme	41.9 ± 7.2	75.8 ± 27	32.1 ± 2.3	–	–	–
CalA	–	72.1 ± 20.1	8.8 ± 0	5.1 ± 0	–	49.1 ± 4
CalB	–	–	–	–	–	–
Lipase *T. lanuginosus*	–	8.6 ± 2.5	–	–	–	35.9 ± 0.4
Lipase *R. miehei*	59.8 ± 13.7	33.6 ± 17.9	7 ± 6.3	3.7 ± 0	–	64.3 ± 10.5
Lipase *C. rugosa*	–	11 ± 12.3	–	–	–	–

The results from the experiments with methyl palmitate clearly differed from those using methyl octanoate. In contrast to the former experiments, transesterification activities of these lipases were observed in MTBE when employing the longer-chained methyl palmitate. A possible explanation might be the better solubility of the more hydrophobic fatty acid in this rather unpolar solvent, which favors its transesterification. Similar to the experiments with methyl octanoate, synthetic activities could be shown for various lipases in 2M2B and *tert*-butanol. These two solvents thus seem capable of dissolving fatty acids with short to medium chain length as well as long chained fatty acids. In the rather polar solvents, 2-propanol and acetone, almost no activity could be detected when using methyl palmitate. The highest transesterification activities were measured when using Novozyme 435 in MTBE and 2M2B. Additionally the combination of Lipozyme or Cal A in 2M2B resulted in relatively high transesterification activities.

Thus, the pNP assay can monitor the transesterification activities of different lipase-solvent combinations. The results with the immobilized lipases showed the highest activities as they exhibit enhanced stability and activity when employed in organic solvents. To validate these findings,and in order to investigate the suitability of the lipase-solvent combinations for an enzymatic glycolipid synthesis, further experiments were performed using glucose as substrate instead of pNP.

### Glycolipid synthesis in organic solvents

All possible combinations of lipases and solvents were used in experiments for the synthesis of glycolipids employing glucose and methyl octanoate or methyl palmitate. To qualitatively monitor possible successful glycolipid syntheses all experiments were analyzed via TLC. Comparable experiments showed that glycolipids consisting of one sugar molecule monoacylated with octanoate typically have a retention factor (Rf) of approx. 0.43, while glycolipids containing one sugar molecule monoacylated with palmitate yield Rf values of approximately 0.56.

In all experiments, the results varied between whether glycolipid synthesis occurred or did not. Identical results were observed using methyl octanoate and methyl palmitate and the results of all TLC analyses are summarized in Table 3.

**Table 3:** Results of the TLC analysis of the lipase-catalyzed synthesis of glucose octanoate (Rf value of 0.43) or glucose palmitate (Rf value of 0.56)

Bands detected by TLC	MTBE	2M2B	t-Butanol	2-Propanol	Acetone	ACN
Novozyme 435		+	+		+	+
Lipozyme		+	+			+
CalA						+
CalB		+	+		+	+
Lipase *T. lanuginosus*						
Lipase *R. miehei*						
Lipase *C. rugosa*						

The results of the glycolipid syntheses using methyl octanoate and methyl palmitate showed that the use of most enzyme-solvent combinations exhibiting positive results using the pNP assay also led to the formation of glycolipids, including those combinations giving the best results. It was, however, also observed that some lipases, such as the lipase from *T. lanuginosus* or *R. miehei*, which both were used in liquid formulations, did not exhibit any transesterification activity in any solvent tested, although activity in the pNP assay was detected. Also the lipase from *C. rugosa*, which was lyophilized, did not synthesize glycolipids. CalA only showed transesterification activity in acetonitrile, which also depicts a clear difference from the results obtained from the pNP ester synthesis, where a transesterification activity of CalA was observed in 2M2B and 2-propanol when using methyl octanoate. These findings indicate that these lipases might have been able to utilize pNP as a substrate, but not glucose.

In all experiments using MTBE or 2-propanol, glycolipid synthesis was not detected although quite high transesterification activities were detected using the pNP assay. A possible explanation might be the relatively high logP value of MTBE. During the experiments, the employed sugars precipitated and did dissolve properly in nonpolar MTBE. This shows that the possible outcome of a synthesis with a distinct solvent—lipase combination can hardly be predicted and is likely to be influenced by more parameters than just the solvent polarity. Other factors having an impact on enzyme activity and stability might be the solubility of the substrate, the water activity of the solvent or the presence of functional groups and their conformation in the solvent itself, which affects the solvent enzyme interaction, especially in the active center of the lipase. Also conformational changes of the enzyme might occur and thereby lead to a shift or loss of activity or even denaturation of the enzyme. This emphasizes the utility of a small-scale assay to directly measure the lipase activity in the employed solvent.

The solvents providing the best combination of substrate solubility and enzyme stability and activity are 2M2B, *tert*-butanol, acetone and acetonitrile. Especially the lipase B from *C.* exhibited positive results, with Novozyme 435 (immobilized Lipase B) and CalB (free, lyophilized Lipase B) being able to synthesize glycolipids in four different solvents. Further the other immobilized lipase tested, Lipozyme, was able to synthesize glycolipids in 2M2B, *tert*-butanol and acetonitrile.

The fact that the same combinations of enzymes and solvents led to the formation of glycolipids proved that the lipases from *C. antarctica* (Novozyme 435 and CalB) and Lipozyme are able to accept fatty acids with medium chain lengths as well as fatty acids with longer chain lengths as substrates for a transesterification to yield glycolipids.

## Conclusions

A microtiter plate-based 4-nitrophenol assay was developed which was suitable to measure the transesterification activity of lipases in organic solvents in a time-saving and high throughput manner. In addition to already known assays, this assay allows the use of less nonpolar solvents also suitable for glycolipid syntheses, allowing the effective screening of enzyme-solvent combinations to fit this cause. The use of a small scale assay measuring the desired enzyme activity via a color reaction accelerates and facilitates the selection of enzyme-solvent combinations, since time consuming methods to detect the formation of transesterification products such as GC or HPLC, can be avoided. Furthermore the described assay might be used to characterize other enzymes with unknown transesterification activities in organic solvents.

## Abbreviations

2M2B: 2-Methyl-2-butanol
ACN: Acetonitrile
MeOOct: Methyl *n*-octanoate
MeOPalm: Methyl *n*-palmitate
MTBE: Methyl *tert*-butyl ether
pNP: 4-Nitrophenol
Rf: Retention factor
